# Supplementary material for: Genetic comparisons of interleukin-17 reveal a framework for complex signaling evolution
Source: bioRxiv. 2026 Apr 14:2026.04.13.718218. Preprint. [Version 1] doi: 10.64898/2026.04.13.718218 (PMC13105070; doi:10.64898/2026.04.13.718218)
Supplement: Supplement 3 [file media-3.zip › Supplemental files for paper/ERC/IL17xIL17_comparisons/IL25_IL17RE.pdf]

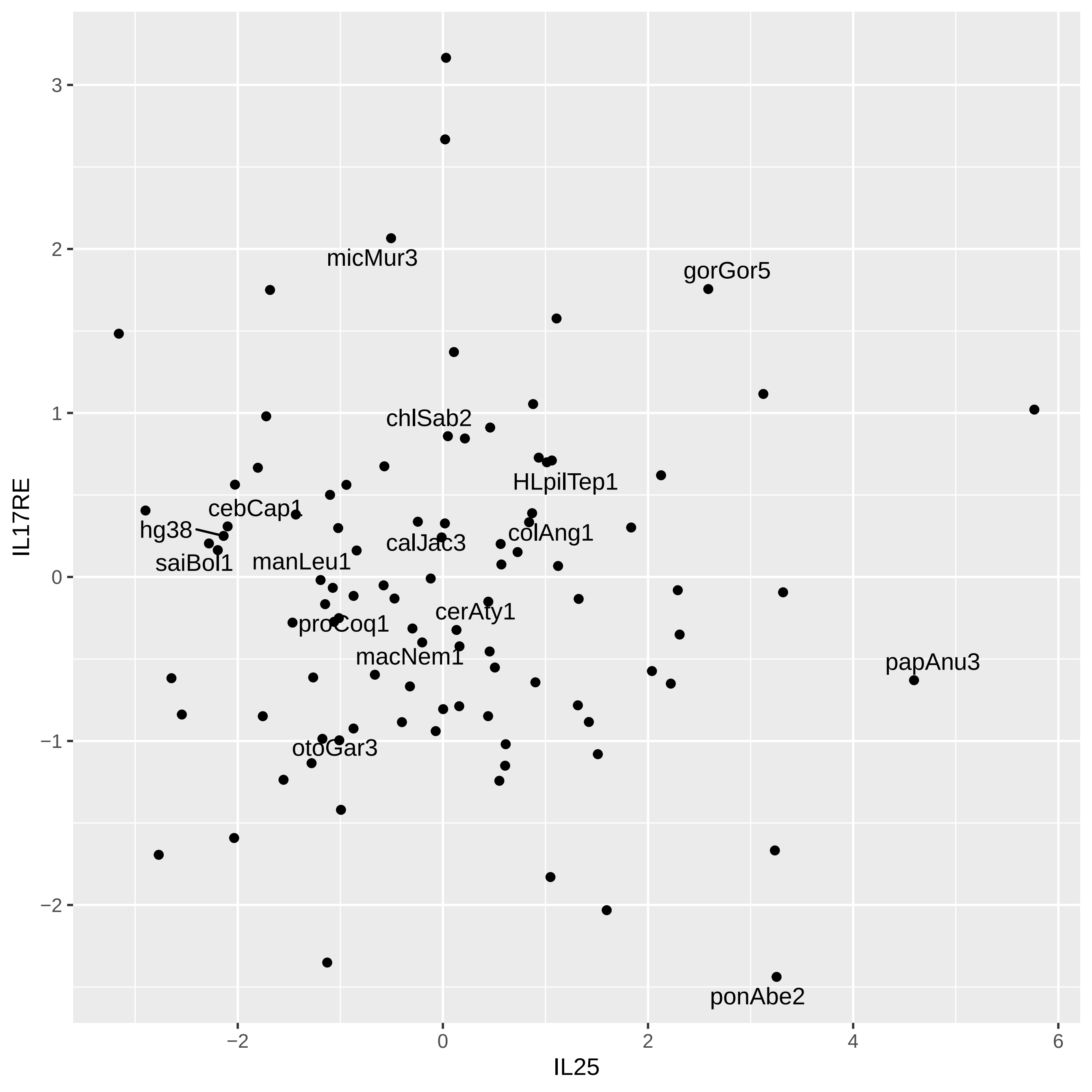

IL17RE
IL25
micMur3
gorGor5
chlSab2
HLpilTep1
cebCap1
hg38
colAng1
calJac3
saiBol1
manLeu1
cerAty1
proCoq1
macNem1
papAnu3
otoGar3
ponAbe2
3
2
1
0
−1
−2
−2
0
2
4
6
